# Temperature-modulated separation of vascular cells using thermoresponsive-anionic block copolymer-modified glass

**DOI:** 10.1016/j.reth.2024.03.009

**Published:** 2024-04-06

**Authors:** Tadashi Hirotani, Kenichi Nagase

**Affiliations:** a Faculty of Pharmacy, Keio University, 1-5-30 Shibakoen, Minato-ku, Tokyo, 105-8512, Japan; b Graduate School of Biomedical and Health Sciences, Hiroshima University, 1-2-3 Kasumi, Minami-ku, Hiroshima, 734-8553, Japan

**Keywords:** Vascular tissue engineering, Thermoresponsive polymer, Cell separation, Endothelial cells, Smooth muscle cells

## Abstract

**Introduction:**

Vascular tissue engineering is a key technology in the field of regenerative medicine. In tissue engineering, the separation of vascular cells without cell modification is required, as cell modifications affect the intrinsic properties of the cells. In this study, we have developed an effective method for separating vascular cells without cell modification, using a thermoresponsive anionic block copolymer.

**Methods:**

A thermoresponsive anionic block copolymer, poly(acrylic acid)-*b*-poly(*N*-isopropylacryl-amide) (PAAc-*b*-PNIPAAm), with various PNIPAAm segment lengths, was prepared in two steps: atom transfer radical polymerization and subsequent deprotection. Normal human umbilical vein endothelial cells (HUVECs), normal human dermal fibroblasts, and human aortic smooth muscle cells (SMCs) were seeded onto the prepared thermoresponsive anionic block copolymer brush-modified glass. The adhesion behavior of cells on the copolymer brush was observed at 37 °C and 20 °C.

**Results:**

A thermoresponsive anionic block copolymer, poly(acrylic acid)-*b*-poly(*N*-isopropylacrylamide) (PAAc-*b*-PNIPAAm), with various PNIPAAm segment lengths was prepared. The prepared copolymer-modified glass exhibited anionic properties attributed to the bottom PAAc segment of the copolymer brush. On the PAAc-*b*-PNIPAAm, which had a moderate PNIPAAm length, a high adhesion ratio of HUVECs and low adhesion ratio of SMCs were observed at 37 °C. By reducing temperature from 37 °C to 20 °C, the adhered HUVECs were detached, whereas the SMCs maintained adhesion, leading to the recovery of purified HUVECs by changing the temperature.

**Conclusions:**

The prepared thermoresponsive anionic copolymer-modified glass could be used to separate HUVECs and SMCs by changing the temperature without modifying the cell surface. Therefore, the developed cell separation method will be useful for vascular tissue engineering.

## Highlights

• Thermoresponsive anionic block copolymer-modified glass was developed.
• HUVECs exhibited a high cell adhesion ratio on the prepared copolymer.
• SMCs exhibited a low cell adhesion ratio on the prepared copolymer.
• The copolymer-modified glass separated HUVEC and SMC by changing the temperature.
• The copolymer-modified glass is useful for vascular tissue engineering.

## Graphical abstract

**Figure:**
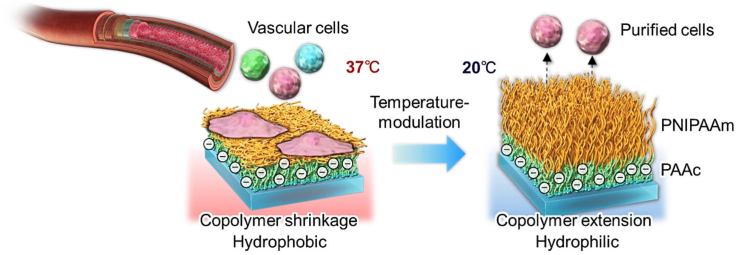

## 1 Introduction

In recent decades, regenerative therapies involving the transplantation of cell suspensions or tissues have emerged promising for treating intractable disease [[1], [2], [3], [4], [5], [6]]. Vascular tissue engineering is the key technology [[7], [8], [9], [10], [11]]. Various types of vascular tissues have been developed *in vitro* from vascular endothelial cells and smooth muscle cells using various tissue engineering technologies [3,8,[12], [13], [14]]. In addition, vascularization of cellular tissues is required to supply oxygen and nutrients [[15], [16], [17], [18], [19], [20], [21]].

In tissue engineering, separation of vascular cells is a crucial step in preparing cell suspensions and constructing cellular tissues. For example, when collecting cells from living tissues, different cell types often mix. To ensure successful transplantation or cellular tissue fabrication, the target cells must be purified from other cell types. Several cell separation techniques have been developed to achieve this goal [[22], [23], [24], [25], [26], [27], [28]]. Cell separation methods involving modification of cells with fluorescently labeled antibodies or antibodies conjugated to magnetic particles are commonly employed. However, cell modification techniques can affect the intrinsic properties of cells, potentially compromising their therapeutic effectiveness or efficiency in constructing cellular tissues. Therefore, innovative methods are required to separate cells without modifying their surfaces or altering their properties.

Cell separation using poly(*N*-isopropylacrylamide) (PNIPAAm) is a non-modifying technique [[29], [30], [31], [32], [33], [34]]. PNIPAAm exhibits thermo-responsive characteristics that are influenced by hydration and dehydration across its phase transition temperature [35]. PNIPAAm undergoes changes in both its hydrophilic and hydrophobic properties near the phase-transition temperature. Moreover, PNIPAAm exhibits extension and shrinkage below and above the transition temperature, respectively. The unique properties of PNIPAAm have been applied in various biomedical contexts, such as temperature-regulated drug and gene delivery systems [[36], [37], [38], [39], [40], [41]], bioanalysis and biosensor devices [[42], [43], [44], [45], [46], [47]], nano-actuators [[48], [49], [50], [51]], bioseparation tools [[52], [53], [54], [55], [56], [57], [58], [59], [60], [61], [62], [63]], cell-separation materials [[32], [33], [34],[64], [65], [66], [67], [68], [69], [70]], cell culture substrates [[71], [72], [73], [74], [75], [76], [77]], and cell sheet therapy in diverse types of regenerative medicine [5,[78], [79], [80], [81], [82], [83], [84], [85], [86], [87], [88]]. Temperature-controlled cell adhesion and detachment were applied to cell separation procedures using PNIPAAm-modified substrates. PNIPAAm exhibits hydrophobic properties at 37 °C due to dehydration, leading to the increased adhesion of cells to PNIPAAm-modified surfaces at this temperature. Conversely, when the temperature was reduced to 20 °C, PNIPAAm became hydrophilic, resulting in poor cell adhesion. Moreover, on PNIPAAm-modified substrates, cell adhesion and detachment behaviors differed among cell types, leading to cell separation. For example, endothelial cells and myoblasts adhere to PNIPAAm-modified substrates at 37 °C, However, endothelial cells rapidly detach from the surface when the temperature is reduced to 20 °C, while myoblasts detach more slowly [30]. Thus, endothelial cells and myoblasts recover after the initial incubation period [30].

The incorporation of ionic groups into PNIPAAm is an effective approach for increasing the differences in cell adhesion behavior as cells have different electrostatic properties, which leads to variations in adhesion behavior [64,65,69,89,90]. In addition, smooth muscle cell adhesion is enhanced by the carboxyl group of the PNIPAAm-modified substrate [91].

Block copolymers composed of upper thermoresponsive polymer segments and lower cell-affinity polymer segments on substrates have proven to be effective polymer-modified structures for temperature-modulated cell separation [67,70]. In the polymer-modified substrate, the upper PNIPAAm dehydrates and shrinks at 37 °C, leading to cell adhesion attributed to the enhanced interaction between cells and the lower affinity polymer segment. By reducing temperature to 20 °C, the upper PNIPAAm hydrates were extended, leading to cell detachment attributed to the reduced interaction between cells and the lower affinity polymer segment.

**Fig. 1:**
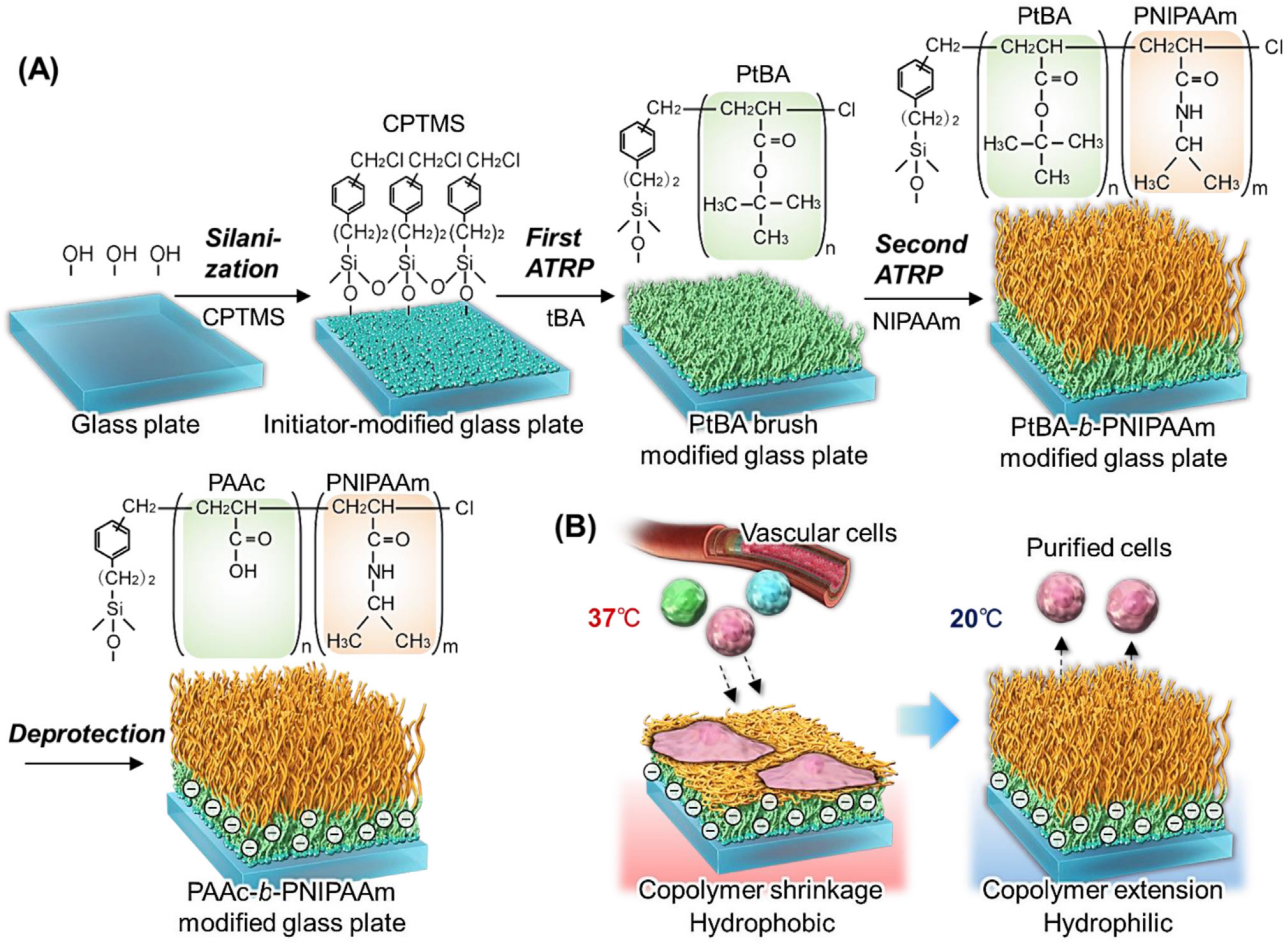
Schematic of temperature-modulated vascular cell separation using a thermoresponsive anionic block copolymer brush. (A) Preparation scheme for thermoresponsive-anionic block copolymer, PAAc-*b*-PNIPAAm, brush-modified glass through two atom transfer radical polymerization (ATRP) steps and deprotection of the *tert*-butyl group; (B) Temperature-modulated vascular cell separation.

Based on these previous reports, a block copolymer brush composed of an upper thermoresponsive polymer segment and a lower anionic polymer segment would be a great candidate for an effective vascular cell separation material because the affinity between vascular cells and carboxyl groups can be modulate by temperature change. A previous report suggested that the carboxyl-group-introduced copolymer P(NIPAAm-*co*-acrylic acid (AAc)-*co*-*tert*-butyl acrylamide (tBAAm)) exhibited effective adhesion to vascular cells [65]. In addition, the block copolymer brush P(*N,N*-dimethyl aminopropyl acrylamide (DMAPAAm)-*b*-PNIPAAm exhibited an effective cell separation performance in a previous study [64]. In this study, we developed a thermoresponsive anionic block-copolymer brush-modified glass substrate for cell separation (Fig. 1). Cell separation performance was evaluated by observing temperature-modulated vascular cell adhesion.

## 2 Materials and methods

### 2.1 Preparation of thermoresponsive anionic block copolymer brush

Details of the reagents, cells, and cell culture media used in this study are provided in the Supplementary Material.

A thermoresponsive anionic copolymer brush was prepared through two-step atom transfer radical polymerization (ATRP) to generate a temperature-modulated cell separation substrate (Fig. 1).

Glass cover slips (24 mm × 50 mm, thickness: 0.17–0.25 mm) were placed in a glass holder, and their surface was cleaned using a plasma gas cleaner (PM100; Yamato, Tokyo, Japan). The glass holder containing the slips was then placed in a 500 mL separable flask at 60% relative humidity and 25 °C for 2 h (Chloromethyl)phenylethyl) trimethoxysilane (CPTMS) (3.54 mL) was dissolved in toluene (300 mL) and the CPTSM solution was poured into the flask. The silane coupling reaction was carried out at 25 °C for 18 h to modify the glass surface using CPTMS. After rinsing the glass slips with toluene and methanol, they were dried at 110 °C for 3 h under vacuum.

The first ATRP was performed to modify poly(*tert*-butylacrylate)(PtBA) on CPTMS-modified glass. *tert*-Butylacrylate (tBA; 3.80 g, 128 mmol) was dissolved in 300 mL 2-propanol. The tBA solution was deoxygenated by bubbling it with Ar gas for 30 min CuCl_2_ (8.07 mg, 0.0600 mmol), and ascorbic acid (106 mg, 0.602 mmol) were added to the solution, and argon gas was bubbled through the solution for 30 min. Further, tris[(2-dimethylamino)ethyl]amine (Me_6_TREN) (138 mg, 0.599 mmol) was added to the solution. The flask was then sealed and placed in a glove bag. CPTMS-modified glass slips with a glass holder in a separable flask were placed in the same glove bag. Oxygen in the glove bag was removed under vacuum with flowing Ar gas. The tBA solution was poured into the separable flask, and *α*-chloro-*p*-xylene (26.3 μL, 0.198 mmol) was added to the reaction solution. The separable flask was sealed and the ATRP reaction proceeded at 25 °C for 1 h. After the reaction, the glass slips were rinsed and washed with methanol in an ultrasonic bath for 30 min. The glass was dried under vacuum at 50 °C.

A second ATRP step was conducted to modify the PNIPAAm on the PtBA-modified glass. NIPAAm was dissolved in 2-propanol (300 mL) at various NIPAAm concentrations (500, 750, and 1000 mM) to modulate the PNIPAAm segment length. NIPAAm (16.9 g, 0.15 mol) was dissolved in a mixed solvent to prepare a 500 mM NIPAAm solution. The NIPAAm solution was deoxygenated by bubbling it with Ar gas for 30 min CuCl_2_ (8.07 mg, 0.0600 mmol), and ascorbic acid (106 mg, 0.602 mmol) were added to the solution, and argon gas was bubbled through the solution for 30 min. Subsequently, Me_6_TREN (138 mg, 0.599 mmol) was added to the solution. The flask was then sealed and placed in a glove bag. The PtBA-modified glass slips with a glass holder in a separable flask were placed in the same glovebag. The glove bag was deoxygenated by bubbling vacuum and argon gas. The NIPAAm solution was poured into the separable flask, and *α*-chloro-*p*-xylene (26.3 μL, 0.198 mmol) was added to the reaction solution. The flask was sealed and the reaction proceeded at 25 °C for 16 h. After the reaction, the glass slips were rinsed with acetone to and the PtBA-*b*-PNIPAAm-modified glass was obtained.

The *tert*-butyl groups were deprotected by immersing the PtBA-*b*-PNIPAAm-modified glass slips in a 5% methanesulfonic acid/dichloromethane solution for 1 h. After deprotection, the glass slips were rinsed with methanol and dried at 50 °C for 3 h, and polyacrylic acid (PAAc)-*b*-PNIPAAm-modified glass was obtained. The PAAc-*b*-PNIPAAm-modified glass was named “PAAc-PN- X,” where X is the NIPAAm monomer concentration.

### 2.2 Characterization of thermoresponsive anionic block copolymer brush and cells

The thermoresponsive anionic block copolymer-modified glass was characterized by measuring its surface zeta potential using gel permeation chromatography (GPC). The zeta potentials of the copolymer-modified surfaces were measured using a zeta potential analyzer (ELSZ-2000SZ; Otsuka Electronics, Osaka, Japan). The copolymer-modified glass was cut into 15 × 35 mm pieces, and the cut glass was attached to the cell for a flat sample of the zeta potential analyzer. The monitored particles were suspended in a NaCl solution (10 mM). The particle suspension was poured into copolymer-modified glass-attached cells. The zeta potential of the copolymer-modified glass was observed at 37 °C. The molecular weight of the prepared polymer in the ATRP was determined using GPC (HLC-8020GPC; Tosoh, Tokyo, Japan). Two serially connected GPC columns (TSK-GEL α-M, Tosoh) were used. *N,N*-Dimethylformamide with 50 mM lithium chloride was used as the mobile phase at a flow rate of 1.0 mL/min. The polymer elution from the column was observed using a refractive index detector. The molecular weight was determined using a calibration curve created using a polyethylene glycol standard. The zeta potentials of normal human umbilical vein endothelial cells (HUVECs), normal human dermal fibroblasts (NHDFs), and human aortic smooth muscle cells (SMCs), were determined using a zeta potential analyzer (ELSZ-2000SZ). A 10 mM phosphate buffer solution (pH 7.4) was prepared by mixing sodium dihydrogen phosphate and disodium hydrogen phosphate. Sucrose was dissolved 0.25 mol/L in the solution to make it isotonic. The cells were then diluted to a density of 1 × 10^5^ cells/mL and the zeta potential was measured at 37 °C using a zeta potential analyzer.

### 2.3 Temperature-modulated selective vascular cell adhesion and detachment

Temperature-modulated cell adhesion and detachment on the prepared PAAc-*b*-PNIPAAm-modified glass were observed to investigate its suitability as a cell separation material. HUVECs, NHDFs, and SMCs were used as model cells for cell separation in vascular tissue engineering.

The copolymer-modified glass slips were cut into 24 mm × 25 mm pieces and placed in a 35 mm polystyrene dish. The copolymer-modified glass in the dish was sterilized using 70% ethanol and UV light irradiation. The glass plates were rinsed with phosphate-buffered saline.

Cell suspensions (5 × 10^4^ cells/mL) of HUVECs, NHDFs, and SMCs were prepared using cell culture media for each cell type (Table S1) and 2 mL of the suspension was seeded onto the copolymer-modified glass in the dish. The dish was then incubated at 37 °C for 24 h in a CO_2_ incubator (9100EX; Waken Btech, Kyoto, Japan). At predetermined time points during the incubation, cell adhesion on the copolymer-modified glass was observed using a phase-contrast microscope (BZ-X800; Keyence, Osaka, Japan). The cell adhesion rate was estimated as the ratio of the number of adhered cells observed in the microscopic images to the number of seeded cells (1 × 10^5^ cells/dish). After incubation at 37 °C for 24 h, the dish was placed in a CO_2_ incubator (9100EX) at 20 °C for 3 h. At predetermined time points during incubation, cell adhesion was observed using phase-contrast microscopy and the cell adhesion ratio was calculated. For comparison, cell adhesion behavior on TCPS was also observed.

In the cell separation experiment, SMCs and HUVECs were stained red and green, respectively, using cell-staining reagents. The cell suspensions were mixed in a 1:1 ratio and 2 mL of the cell suspension (5 × 10^4^ cells/mL) was seeded onto a PAAc-*b*-PNIPAAm-modified glass plate (PAAc-PN750) in a dish. The dishes were incubated at 37 °C for 24 h. During incubation, cell adhesion to the copolymer-modified glass was observed at predetermined times using a fluorescence microscope (BZ-X800; Keyence). The cell adhesion ratio was defined as the ratio of the number of adhered cells observed in the microscopic images to the number of seeded cells. The dish was then incubated at 20 °C for 3 h. Cell adhesion on the copolymer-modified glass during incubation was observed by fluorescence microscopy at predetermined times.

## 3 Results and discussion

### 3.1 Characterization of the thermoresponsive anionic block copolymer brush and cells

Thermoresponsive anionic block copolymer brushes were prepared using a two-step ATRP method. The polymer prepared in the reaction solution during the second ATRP was characterized using GPC to investigate the PNIPAAm segment length of PAAc-*b*-PNIPAAm on glass (Table 1). The code of PNIPAAm prepared second ATRP was “PN-X,” where X is the NIPAAm monomer concentration. The molecular weight of PNIPAAm increased with increasing NIPAAm monomer concentration during the ATRP, which was consistent with previous reports [64]. This is because the ATRP polymerization rate increases with increasing monomer concentration. Similarly, the length of the PNIPAAm segment increases with increasing NIPAAm concentration. PN-1000 exhibits a significantly higher molecular weight than PN-500 and PN-750. This could be attributed to the reduced control of ATRP at elevated monomer concentrations. In fact, higher polydispersity was observed in PN-1000 than that in PN-500 and PN-750, indicating that the polymerization control of PN-1000 was relatively weak compared to that of PN-500 and PN-750.

**Table 1:** Characterization of the prepared PNIPAAm in the second ATRP.

Code^a^	Monomer concentration (mM)	*M*_*n*_^b^	*M*_*w*_*/M*_*n*_^b^
PN-500	500	7300	1.3
PN-750	750	8900	1.2
PN-1000	1000	23,000	1.5

a The code of PNIPAAm prepared second ATRP was “PN-X,” using NIPAAm monomer concentration.

b Determined by gel permeation chromatography using DMF with 50 mM LiCl as the mobile phase.

In addition, we investigated the molecular weight of PAAc. However, PAAc interacts with the GPC column, leading to an uncorrected molecular weight for PAAc. In a previous report, different types of polymer, P(2-hydroxyethyl methacrylate-*co*-propargyl acrylate) was grafted onto glass via the same atom transfer radical polymerization [67]. The molecular weight of the prepared polymer was determined by GPC and the molecular weight was 8400. Thus, similarly, the prepared PAAc in this study would probably be the same.

The zeta potentials of the prepared copolymer brushes are listed in Table 2. The zeta potential of the PAAc-modified glass indicated strong anionic properties because of the carboxyl groups of PAAc on the glass. The PAAc-*b*-PNIPAAm brush-modified glasses, PAAc-PN-500, PAAc-PN-750, and PAAc-PN-1000, exhibited relatively weak anionic properties compared with those of PAAc because of the modification of PNIPAAm on PAAc. PAAc-*b*-PNIPAAm was prepared by modifying the PNIPAAm segment. Thus, the lower PAAc segment was concealed from the upper PNIPAAm segment, weakening the surface anionic properties of the copolymer-brush-modified glass. Similar negative zeta potentials were observed in PAAc-PN-500, PAAc-PN-750, and PAAc-PN-1000, ranging from −3.26 to −1.31. These results indicated that the PNIPAAm segment length did not influence the zeta potential of the glass, probably because of the sufficient anionic coverage of PAAc with PNIPAAm.

**Table 2:** Zeta potentials of the prepared copolymer-modified glass.

Code	Monomer concentration (mM)	Zeta potential (mV)^a^
PAAc	100	−18.8
PAAc-PN-500	500	−2.33
PAAc-PN-750	750	−1.31
PAAc-PN-1000	1000	−3.26

a Determined using a zeta potential analyzer with 10 mM sodium chloride.

In addition, we intended to observe the zeta potential of the copolymer brush at 20 °C. However, we could not measure the zetapotential at 20 °C, probably because the leakage of the dispersion of the monitor beads attributed to the reduced cohesiveness of the attachment of the measurement cell. Previous reports indicated that ionic block copolymer brushes exhibited reduced ionic properties with decreasing temperature, which was attributed to the concealed bottom ionic segment [54,55]. Similarly, the prepared block polymer brushes might reduce the anionic property at 20 °C compared to that at 37 °C.

**Fig. 2:**
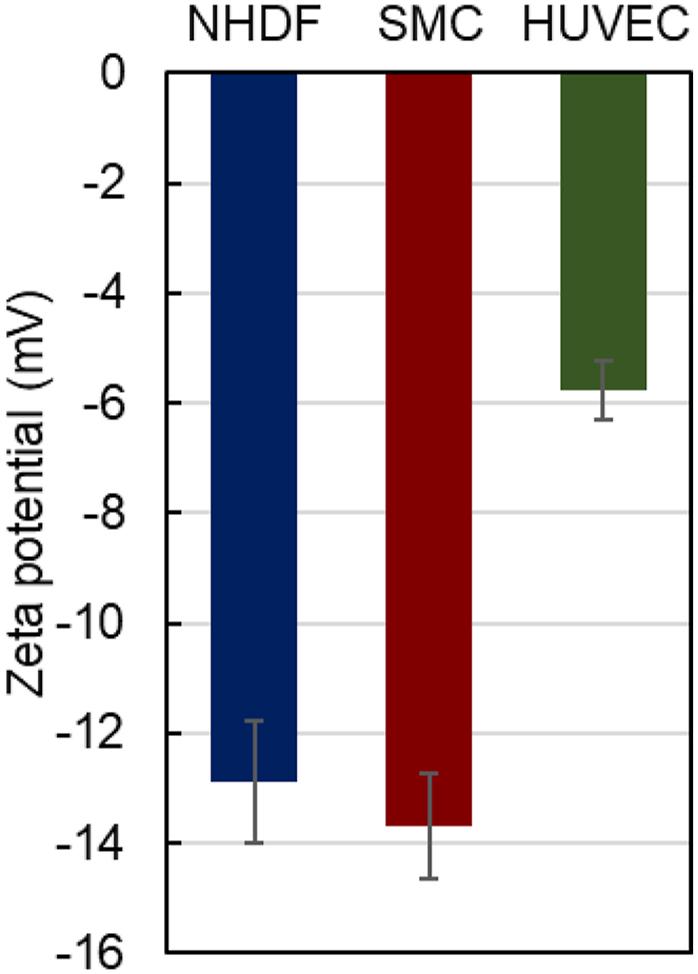
Zeta potentials of normal human dermal fibroblasts (NHDFs), smooth muscle cells (SMCs), and human umbilical vein endothelial cells (HUVECs).

To investigate the electrostatic properties of the cells, the zeta potentials of NHDF, SMC, and HUVEC were measured using a zeta potential analyzer (Fig. 2). NHDFs and SMCs exhibited a strong negative zeta potential compared to HUVECs. This is probably because differences in cell membrane proteins and sugar chains on the cell surfaces lead to differences in the electrostatic properties of the cells.

### 3.2 Temperature-modulated cell adhesion on the copolymer brush

**Fig. 3:**
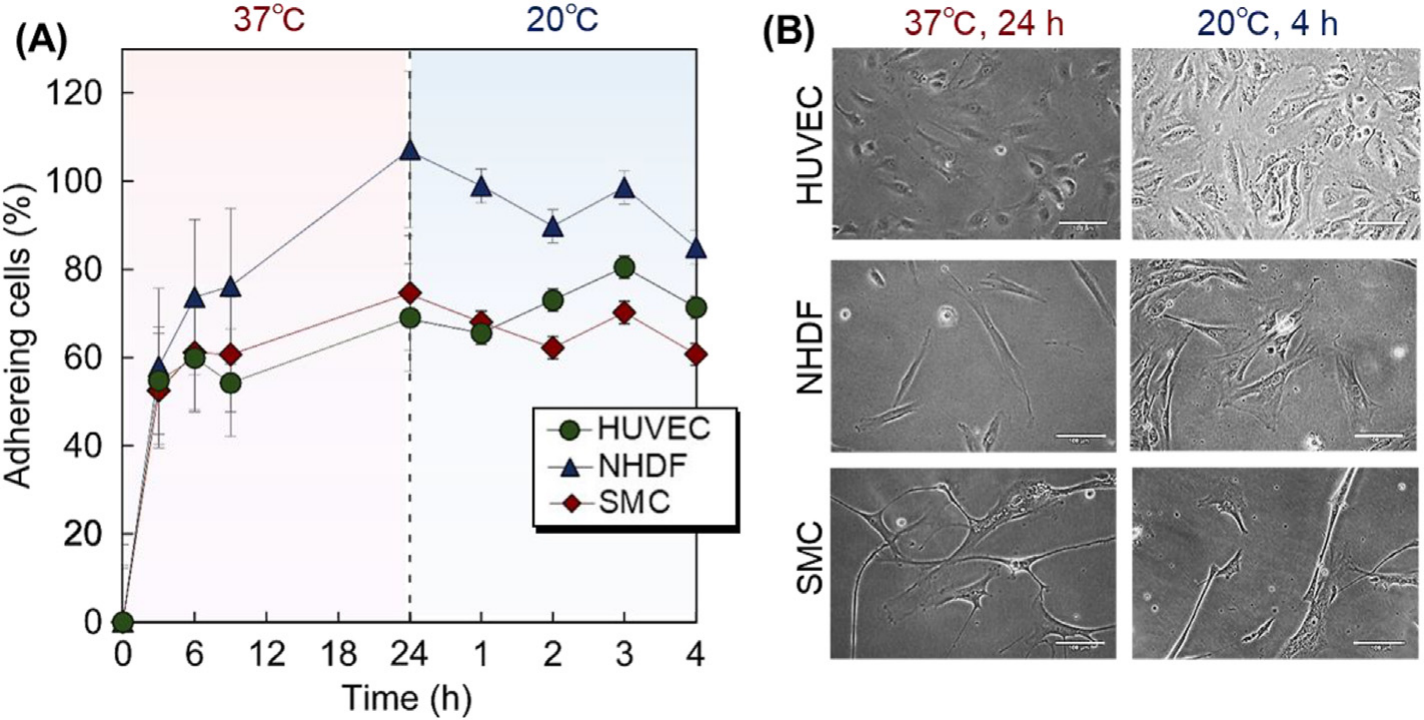
Vascular cell adhesion behavior on tissue culture polystyrene. (A) Cell adhesion profiles during incubation at 37 °C and 20 °C. (B) Cell morphology on tissue culture polystyrene plates. Scale bar: 100 μm. HUVEC, human umbilical vein endothelial cell; NHDF, normal human dermal fibroblast; SMC, smooth muscle cell.

**Fig. 4:**
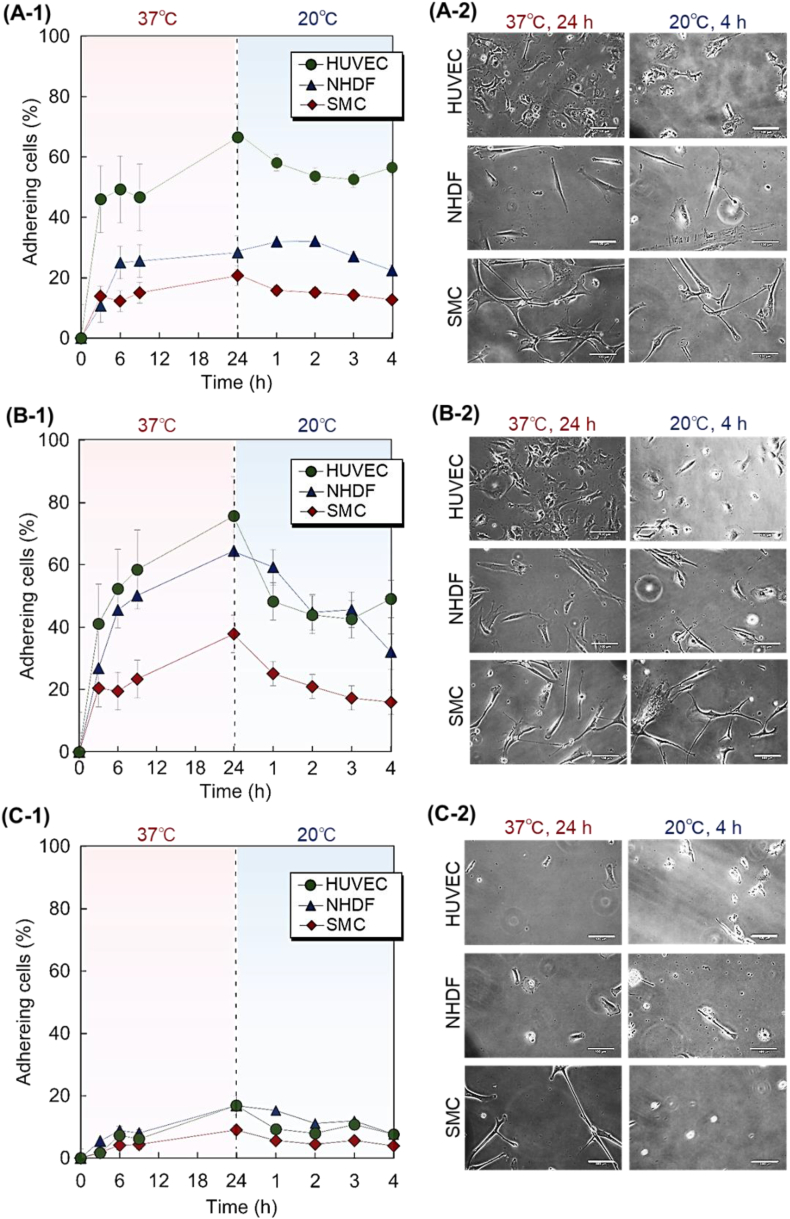
Vascular cell adhesion behavior on PAAc-b-PNIPAAm modified glass substrates. (A) PAAc-PN-500, (B) PAAc-PN-750, and (C) PAAc-PN-1000. (1) Cell adhesion profiles during incubation at 37 °C and 20 °C. (2) Cell morphology on the PAAc-PN-500 copolymer-modified glass substrate. Scale bar: 100 μm. HUVEC, human umbilical vein endothelial cell; NHDF, normal human dermal fibroblast; SMC, smooth muscle cell.

To investigate the availability of the prepared thermoresponsive copolymer brushes for cell-separation materials, the cell adhesion behavior on the copolymer brush at 37 °C and 20 °C was investigated (Fig. 3, Fig. 4). Three types of vascular cells, HUVECs, NHDFs, and SMCs, were seeded on the copolymer brush and cell adhesion on the prepared copolymer brush during incubation at 37 °C and 20 °C was observed. For comparison, cell adhesion behavior on TCPS was also observed (Fig. 3).

To investigate the intrinsic adhesion properties of vascular cells, cell adhesion behavior on commercially available TCPS was observed (Fig. 3). NHDF exhibited high adhesion properties after incubation at 37 °C for 24 h compared with HUVECs and SMCs. This was due to the relatively strong adhesion and proliferative abilities of NHDFs. Previous reports have indicated that NHDFs exhibit good cell adhesion and proliferation abilities compared with other cell types [34,64,65]. Thus, NHDFs adhered to TCPS and slightly proliferated during incubation at 37 °C for 24 h, leading to a cell adhesion ratio exceeding 100%. The other two cell types also adhered to TCPS, although the cell adhesion ratio was relatively low compared to that of NHDFs. After reducing the temperature to 20 °C, the adhered cells maintained their adhesion even after 4 h of incubation. Cell adhesion appeared to be reduced at 20 °C compared at 37 °C. However, all cell types maintained their adhesion to TCPS. These results indicated that the cells were not detached by simply changing the incubation temperature.

Vascular cell adhesion to the PAAc-PN-500 copolymer-modified glass substrate was also observed (Fig. 4A). On the copolymer brush, a relatively high level of adhesion of HUVECs was observed after incubation at 37 °C for 24 h compared to the adhesion of NHDFs and SMCs. This may be attributed to the weak anionic properties of HUVECs compared with those of NHDFs and SMCs. PAAc-PN-500 The anionic properties attributed to the carboxyl group of the lower PAAc segment. Thus, electrostatic repulsion between PAAc-PN-500 and the strongly negatively charged NHDFs and SMCs led to the suppression of adhesion compared to weakly negatively charged HUVECs. In addition, the lower PAAc segment enhanced the hydration of the upper PNIPAAm segment, leading to the suppression of cell adhesion at 37 °C. By reducing the temperature to 20 °C, adhered HUVECs slightly detached from the copolymer brush, due to the hydration of the PNIPAAm segment at a reduced temperature. Previous reports have demonstrated that cells adhere to a PNIPAAm-modified substrate at 37 °C due to the hydrophobicity of PNIPAAm and detach from a PNIPAAm-modified substrate at 20 °C due to the hydration of PNIPAAm. Similarly, the adhered cells were detached from the copolymer brush at 20 °C due to hydration of the PNIPAAm segment of PAAc-*b*-PNIPAAm. Contrarily, the detachment of NHDFs and SMCs was relatively low because of the short PNIPAAm segments. In previous reports on the preparation of block-copolymer brushes for cell separation, cell detachment was enhanced by increasing the length of the upper PNIPAAm segment, which was attributed to the cell repulsion of the PNIPAAm segment.

A previous report indicated that a PNIPAAm brush without a PAAc segment exhibited similar adhesion to vascular cells [30]. Conversely, PAAc-*b*-PNIPAAm exhibited different cell adhesion behaviors for vascular cells as the incorporated PAAc segment provides different cell adhesive properties. Thus, the PAAc-*b*-PNIPAAm brushes have higher selectivity for vascular cells than the PNIPAAm brushes.

Additionally, vascular cell adhesion to the PAAc-PN-750 copolymer-modified glass substrate was observed (Fig. 4B). The relatively greater adhesion of HUVECs and NHDFs to the copolymer brush was observed after incubation at 37 °C, whereas the SMC adhesion ratio was relatively low. These results indicate that NHDF adhesion to PAAc-PN-750 was enhanced compared to its adhesion to the PAAc-PN-500 copolymer. PAAc-PN-750 had a relatively longer PNIPAAm segment than PAAc-PN-500, which resulted in reduced electrostatic repulsion between the lower PAAc segment and the NHDFs, leading to enhanced adhesion of the NHDFs compared with their adhesion to PAAc-PN-500. The SMC adhesion was also slightly enhanced in PAAc-PN-750 compared to that in PAAc-PN-500. This may be attributed to the reduced electrostatic repulsion between the SMCs and PAAc with a relatively long PNIPAAm segment. Negligible difference was observed between the zeta potentials of PAAc-PN-500 and PAAc-PN-750 (Table 2). However, the electric repulsion was reduced by increasing the length of the upper PNIPAAm segment, which was attributed to the increased distance between the carboxyl group of the bottom segment and the cells.

By reducing the temperature to 20 °C, adhered HUVECs and NHDFs detached from the copolymer brush. The detachment ratios of HUVEC and NHDF were 26.4% and 32.2%, respectively. SMC showed relatively low detachment (21.6 %) compared to HUVEC and NHDF. The modified PNIPAAm segment of PAAc-PN-750 became hydrophilic by reducing the temperature from 37 °C to 20 °C, leading to cell detachment. The cell detachment ratio of PAAc-PN-750 was larger than that of PAAc-PN-500. This is attributed to the relatively large PNIPAAm segment of PAAc-PN-750, which enhanced hydration and cell detachment.

Cell adhesion was also observed on the PAAc-PN-1000 copolymer-modified glass substrate (Fig. 4C). Three types of cells exhibited low adhesion ratio after incubation for 24 h at 37 °C. This was attributed to the long PNIPAAm segment of PAAc-PN-1000. Previous reports indicated that the hydration of the outermost region of the PNIPAAm brush is enhanced by increasing the molecular weight of PNIPAAm, which was attributed to the increased mobility of the PNIPAAm chain in the outermost region [92,93]. The PNIPAAm segment of PAAc-PN-1000 was longer than those of PAAc-PN-750 and PAAc-PN-1000. This long PNIPAAm segment tended to be hydrophilic, even at 37 °C, leading to the suppression of cell adhesion.

These results show that PAAc-PN-750 exhibited large differences in the cell adhesion ratio and temperature-modulated cell recovery compared to the other polymers tested. Thus, PAAc-PN-750 is a suitable temperature-modulated cell separation material.

### 3.3 Temperature-modulated cell separation using a copolymer brush

**Fig. 5:**
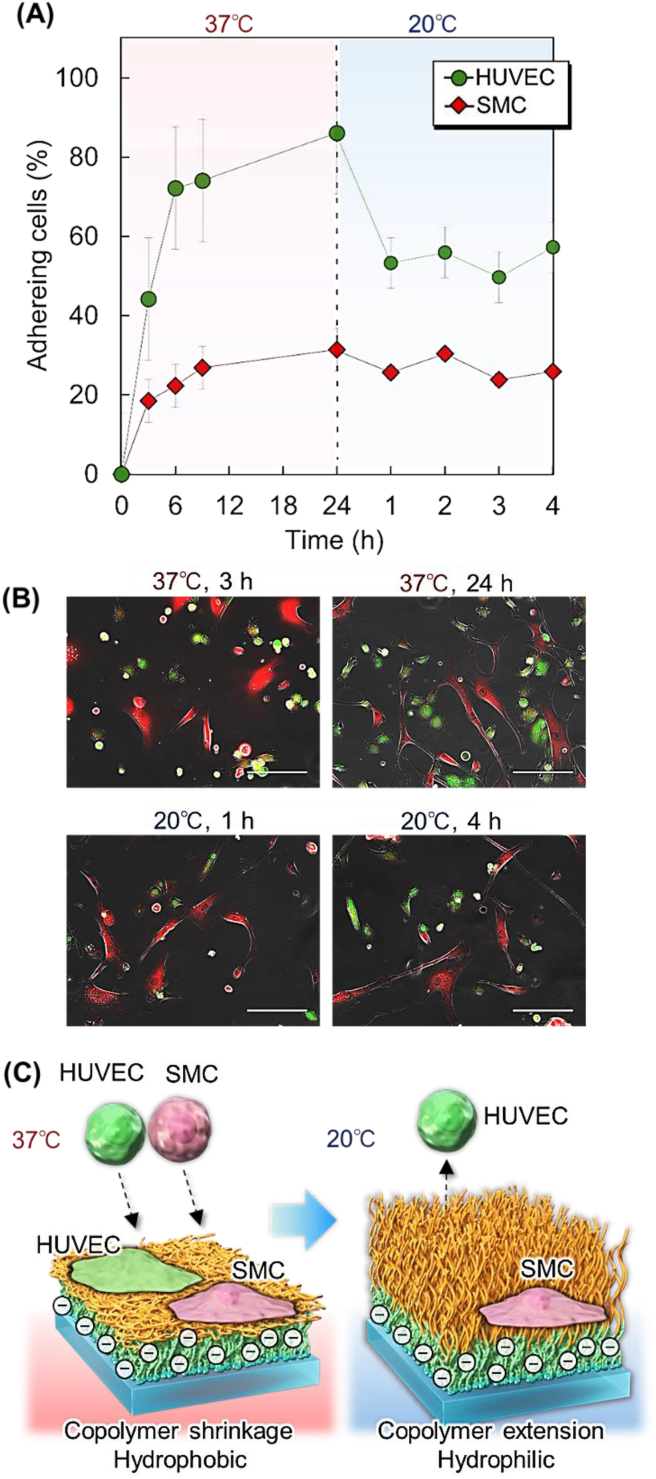
Temperature-modulated cell separation using PAAc-PN-750 copolymer-modified glass substrate. (A) Cell adhesion profiles of mixture human umbilical vein endothelial cells (HUVECs) and smooth muscle cells (SMCs) during incubation at 37 °C and 20 °C. (B) Cell morphology on the PAAc-PN-750-copolymer-modified glass substrate observed by fluorescence microscopy. Green: HUVECs. Red: SMCs. Scale bar: 100 μm. (C) Schematic representation of HUVEC and SMC separation.

The performance of the prepared PAAc-PN-750 as a cell-separation material was investigated. Temperature-modulated cell separation was performed using PAAc-PN-750 and a mixture of HUVECs and SMCs (Fig. 5). HUVECs and SMCs were stained green and red, respectively, using cell-staining reagents. The cells were then mixed in a ratio of 1:1 and seeded onto PAAc-PN-750. The actual HUVEC composition of the blood vessel would be less compared to that of the SMC because the blood vessel is composed of a monolayer of HUVEC and a multilayer of SMC. However, we used a 1:1 composition of HUVEC and SMC in this study as the experiment using a 1:1 composition could identify each cell compared to that using a significantly different composition. After 24 h of incubation at 37 °C, a high adhesion ratio of approximately 90% was observed for HUVECs, whereas SMC adhesion was relatively low (Fig. 5 A and B). By reducing the temperature to 20 °C, adhered HUVECs were detached from PAAc-PN-750, whereas SMCs maintained their adherence to the brush. Previous reports have suggested that cell adhesion behavior differs between coculture and monoculture conditions, which has been attributed to the secreted extracellular matrix under coculture conditions or the reduced activity of cells after the application of cell-staining reagents [70,94]. However, similar cell adhesion behavior was observed in HUVECs and SMCs under both co-culture and monoculture conditions (Fig. 4B). These results indicated that the combination of HUVECs and SMCs did not influence the adhesion behavior of other cell types under co-culture conditions. The HUVECs were able to be captured on a copolymer brush at 37 °C and the captured HUVECs could be recovered by reducing the temperature, resulting in the separation of HUVECs from SMCs (Fig. 5C).

These results indicated that PAAc-*b*-PNIPAAm, with a moderate length of the PNIPAAm segment (PAAc-PN-750), can be used to separate vascular cells simply by changing the temperature from 37 °C to 20 °C. Thus, the developed copolymer brush may be useful as a temperature-modulated cell separation material for vascular tissue engineering.

## 4 Conclusions

Temperature-modulated vascular cell separation materials were developed using a thermoresponsive anionic block copolymer, PAAc-*b*-PNIPAAm, with PNIPAAm segments of various lengths. The thermoresponsive anionic block copolymer was modified on glass substrates using two-step ATRP. The prepared copolymer brushes exhibited weakly anionic properties. HUVECs exhibited relatively weak anionic properties compared to NHDFs and SMCs. A high adhesion ratio of HUVECs was observed on PAAc-*b*-PNIPAAm with a moderately long PNIPAAm segment at 37 °C, whereas the adhesion of SMCs was relatively low. By reducing the temperature from 37 °C to 20 °C, the adhered HUVECs were detached from PAAc-*b*-PNIPAAm, leading to the recovery of HUVECs from the copolymer brush. These results indicate that the developed thermoresponsive anionic block copolymer-modified glass is useful for temperature-modulated separation of vascular cells for vascular tissue engineering.

## Authors contributions

TH and KN conceived of the study. KN supervised the study. TH and KN designed experiments. TH obtained experimental data. TH and KN wrote and reviewed the manuscript.

## Funding

This work was partially supported by Grants-in-Aid for Scientific Research (grant numbers 19H02447, 21KK0199, 22K19899, 20H05233, and 22H04560) from the 10.13039/501100001691Japan Society for the Promotion of Science, Japan, a research grant from CASIO Science Promotion Foundation, and a research grant from the Precise Measurement Technology Promotion Foundation (PMTP-F).

## Declaration of competing interest

The authors declare that they have no known competing financial interests or personal relationships that could have appeared to influence the work reported in this paper.
