# Supplementary material for: Temperature-modulated separation of vascular cells using thermoresponsive-anionic block copolymer-modified glass
Source: Regen Ther. 2024 Apr 6;27:259–67. doi: 10.1016/j.reth.2024.03.009 (PMC11004074; doi:10.1016/j.reth.2024.03.009)
Supplement: Multimedia component 1 [file mmc1.docx]

*Supplementary Materials*

Temperature-modulated cell separation of vascular cells using thermoresponsive-anionic block copolymer-modified glass

Tadashi Hirotani^1^, Kenichi Nagase* ^1,2^

1. Faculty of Pharmacy, Keio University, 1-5-30 Shibakoen, Minato, Tokyo 105-8512, Japan
2. Graduate School of Biomedical and Health Sciences, Hiroshima University, Hiroshima, 734-8553, Japan

*Corresponding author. Tel: +81-3-5400-1378; Fax: +81-3-5400-1378

E-mail: nagase.kenichi@keio.jp, nagase-kn@pha.keio.ac.jp

**S1 Materials**

*N*-isopropylacrylamide (NIPAAm) was obtained from KJ Chemicals (Tokyo, Japan) and recrystallized from *n*-hexane. *tert*-Butylacrylate (tBA) was obtained from Tokyo Chemical Industries (Tokyo, Japan) and purified by distillation before use. Toluene, dichloromethane, 2-propanol, copper (II) chloride (CuCl_2_), ascorbic acid, α-chloro-*p*-xylene, tris(2-aminoethyl)amine (TREN) were obtained from Fujifilm Wako Pure Chemical Corporation (Osaka, Japan). Tris[(2-dimethylamino)ethyl]amine (Me_6_TREN) was synthesized from TREN. ((Chloromethyl)phenylethyl) trimethoxysilane (CPTMS) was purchased from Gelest (Morrisville, PA, USA). Formic acid was obtained from Tokyo Chemical Industries (Tokyo, Japan). Glass cover slips were purchased from Matsunami Glass Industry (Osaka, Japan). Normal human umbilical vein endothelial cells (HUVECs), human aortic smooth muscle cells (AoSMCs), normal human dermal fibroblasts (NHDFs), cell culture media for AoSMCs and NHDFs were purchased from Lonza (Basel, Switzerland). Endothelial cell growth medium was purchased from Promocell (Heidelberg, Germany). Cell staining reagents were obtained from Thermo Fisher Scientific (Waltham, MA, USA). Fetal bovine serum (FBS) was obtained from Biosera (Nuaille, France).

**Table S1** Cell culture media

| Cell line | Cell culture medium ^a)^ | Additives ^b)^ |
| --- | --- | --- |
| Normal human umbilical vein endothelial cell: HUVEC | Endothelial cell basal medium 2 | FCS (10 mL)  hEGF (2.5 μg)  Hydrocortisone (100 μg)  VEGF (0.25 μg)  hbFGF (5 μg)  R3-IGF (1 μg)  Ascorbic acid (500 μg)  Heparin (11.25 mg)  Penicillin-Streptomycin (5 mL) |
| Neonatal normal human dermal fibroblast: NHDF | FBM basal medium | hFGF-β (0.5 mL)  Insulin (0.5 mL)  FBS (10 mL)  Gentamicin/Amphotericin-B (GA) (0.5 mL) |
| Human aortic smooth muscle cells: SMC | Smooth muscle cell basal medium | Insulin (0.5 mL)  hFGF-β (1.0 mL)  GA-1000 (0.5 mL)  FBS (25 mL)  hEGF (0.5 mL) |

a) Volume of cell culture media is 500 mL. b) Additives added to 500 mL of cell culture medium.
